# University for Women in Moscow

**Published:** 1899-11

**Authors:** 


According to the *Argonaut*, a wealthy Russian named Astrokoff, who recently died, left 1,000,000 roubles ($500,000) toward the foundation of a university for women in Moscow, to comprise a mathematic, scientific and medical faculty. The municipal council of Moscow has voted an annual grant of 3,000 roubles ($1,500) to the institution.

